# Social Determinants of Health and Their Association With Parkinson′s Disease Prevalence in US Adults: Insights From NHANES 2001–2020

**DOI:** 10.1155/bn/3745561

**Published:** 2026-06-24

**Authors:** Jinping Fang, Detao Meng, Boyan Fang

**Affiliations:** ^1^ Parkinson Medical Center, Beijing Rehabilitation Hospital, Capital Medical University, Beijing, China, ccmu.edu.cn

**Keywords:** NHANES, Parkinson′s disease, social determinants of health

## Abstract

**Background:**

Social determinants of health (SDoH) have not been comprehensively studied in relation to Parkinson′s disease (PD), although they are well known to be associated with other chronic diseases. Here we assessed the relationship of a multidimensional, comprehensive measure of SDoH with the prevalence of PD in US adults.

**Methods:**

Among 48,637 adults in the National Health and Nutrition Examination Survey (NHANES) between 2001 and 2020, we identified PD cases based on self‐reported antiparkinsonian medication use. We formed a weighted multidomain SDoH score from eight indicators that assessed economic security, education, healthcare access, and social environment. Both multivariable logistic regression and restricted cubic spline (RCS) were used to estimate odds ratios and dose–response relationships and were adjusted for demographic and lifestyle factors.

**Results:**

More intensive adverse SDoH burden was significantly associated with higher PD prevalence (fully adjusted OR 1.31, 95% CI 1.16–1.47 per unit increase), and RCS analysis showed no evidence of nonlinearity (*p* for nonlinearity = 0.370). Unemployment (OR 4.15, 95% CI 2.70–6.40) and food insecurity (OR 1.96, 95% CI 1.39–2.76) were strongly associated with higher PD prevalence, whereas reported lack of health care access was associated with lower identified PD (OR 0.27, 95% CI 0.15–0. 49), pointing to likely ascertainment bias. When stratifying, we observed meaningful effect modification by age (*p* < 0.001), with stronger effects in middle‐aged individuals (≤ 60 years: OR 1.35, 95% CI 1.13–1.61), compared with an attenuation in older individuals.

**Conclusion:**

Poor SDoH were independently associated with higher PD prevalence, particularly among adults aged 60 years and younger. Unemployment and food insecurity showed strong positive associations, whereas the inverse association with healthcare access likely reflects ascertainment bias. These findings are hypothesis‐generating and suggest that SDoH may be relevant to PD screening and epidemiologic research. Longitudinal studies are needed to clarify temporality and causality.

## 1. Introduction

Parkinson′s disease (PD) is among the most common neurodegenerative disorders and represents a substantial and growing public‐health challenge worldwide. The global number of people with PD has risen markedly, from approximately 2.5 million in 1990 to 6.1 million in 2016 (a 2.4‐fold increase), reaching 11.894 million in 2021 and projected to reach 25.224 million by 2050 [1, 2]. The prevalence and incidence of PD rise steeply with increasing age, and PD is associated with substantial disability, decreased quality of life, caregiver burden, and growing demands on health‐care systems. The etiology of PD is complex, with genetic factors and environmental exposures particularly pesticide exposure established as key prevalence contributors. Environmental factors interact with genetics and lifestyle behaviors, jointly influencing the onset and progression of PD [3, 4]. Despite this emphasis on genetic and classical environmental determinants, the contribution of social‐level factors to PD prevalence and progression remains relatively understudied.

Social determinants of health (SDoH) for recognizing health disparities, as seen in the development of public health programs, that is, the US Healthy People 2030 target toward achieving health equity [5, 6]. SDoH refers to characteristics that influence people′s lives and can be organized into areas (e.g., economics stability and health services). They influence health via social–behavioral and biological pathways that influence exposures, health behaviors, care access, and chronic stress [7], contributing to chronic diseases such as cardiovascular disease and diabetes [8–10]. However, evidence relating SDoH to neurodegenerative disorders such as PD is minimal and lacks broad analyses based upon larger datasets.

Prior research investigating SDoH–PD associations is sparse and methodologically restrictive. Most study small regionwide samples, use only income as a measure of one dimension of SDoH or did not sufficiently control for critical covariates such as body mass index (BMI) and lifestyle behavior, which lowers the validity and generalizability of the findings [11, 12]. Furthermore, little research has investigated the additive, multidomain SDoH burden as identified through weighted indices or examined any effect modification by age or BMI, and thus pathways through which SDoH may or may not influence PD prevalence are ill‐defined [13]. Herein, the particular advantages of national health and nutrition examination survey (NHANES) include national representativeness through multistage probability sampling and the prevalence of not just a PD ascertainment but detailed measures for a range of SDoH domains [14]. To build on these strengths and attempt to fill these gaps, the current study uses the NHANES 2001–2020 data to estimate the association of a multidomain combined SDoH score (and its component parts) on PD prevalence, characterize dose–response patterns through restricted cubic splines (RCSs), quantify heterogeneity via stratified and interaction analyses, and determine specific social determinants such as healthcare access and food security that have the strongest effects on PD, thus making explicit what SDoH could mean in terms of independent prevalence factor for PD.

## 2. Methods

### 2.1. Data sources and study populations

**Figure 1:**
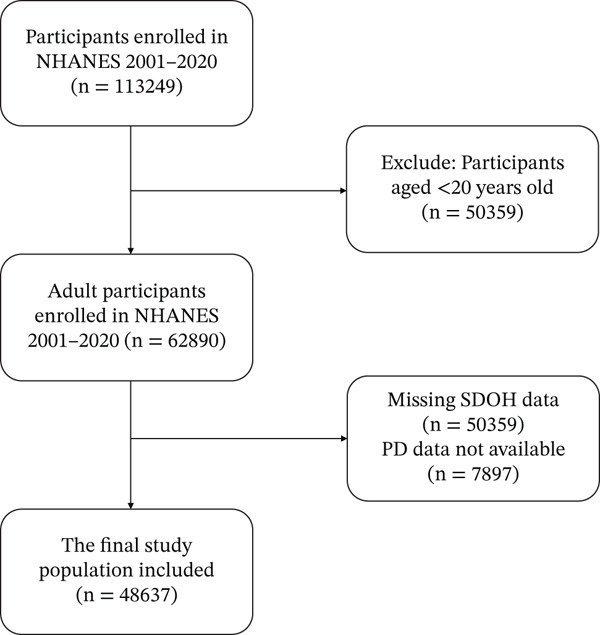
The flow chart of the included participants in this study.

The dataset used for this study is publicly available and can be downloaded from NHANES website (https://www.cdc.gov/nchs/nhanes/index.html). Because the study relied on public domain deidentified databases it did not require ethical review. All subjects provided consent to be involved in collecting NHANES data. NHANES use multistage complex probability sampling to ensure the sampling results represent the general population of the country. All the NHANES study protocols were approved by the Research Ethics Review Board of the National Center for Health Statistics, and the informed consents were obtained for each participating individual. A total of 20‐year (2001–2020) data were collected and analyzed in this cross‐sectional study, the total sample size after exclusion of ineligible participants was 48,637 (Figure 1).

### 2.2. The definition of PD

PD status was operationally ascertained using the NHANES prescription medication data. Because NHANES does not provide a consistently available clinician‐adjudicated PD diagnosis variable across the included survey cycles, participants were classified as having PD if they reported current use of antiparkinsonian medications in the prescription medication files. This approach is consistent with previous NHANES‐based studies that identified PD using antiparkinsonian medication use or prescriptions categorized as antiparkinson agents [15, 16]. Participants who did not report the use of antiparkinsonian medications were classified as non‐PD. This case definition should be interpreted as an operational ascertainment of PD status in NHANES rather than a clinician‐confirmed diagnosis.

### 2.3. SDoH

Data were gathered utilizing a standardized questionnaire, which was administered through direct interviews. The eight SDoH indicators were selected a priori based on the Healthy People 2030 framework and previous NHANES‐based SDoH studies [8, 17]. In accordance with the framework comprising five domains presented by Healthy People 2030 [18], we incorporated eight available NHANES indicators of SDoH: employment status (categorized as employed, student, or retired versus unemployed), the family income‐to‐poverty ratio (≥ 300% compared to < 300%), food security (full security as opposed to marginal, low, or very low security), education level (high school graduate or above versus less than high school), access to healthcare (having at least one regular healthcare facility vs. none or relying solely on emergency departments), health insurance status (private insurance vs. government or uninsured), home ownership (ownership of a home versus renting or other arrangements), and marital status (married or cohabiting with a partner vs. single or not in a partnership) [19].

A weighted composite SDoH score was constructed for the present analysis using a previously described weighting strategy in epidemiological studies [8], rather than a PD‐specific externally validated score. Each SDoH indicator was classified into two categories: advantaged and disadvantaged, with advantaged status coded as 0 and disadvantaged status coded as 1. The *β* coefficients for each SDoH indicator, derived from comparisons between the disadvantaged and advantaged groups in Cox regression models for all‐cause mortality, were used as weights. These models were adjusted for various confounding factors, including age, sex, smoking habits, alcohol consumption, dietary patterns, physical activity (PA), BMI, hypertension, diabetes, cardiovascular diseases, and cancer. The weighted composite SDoH score was calculated by multiplying each binary SDoH indicator by its corresponding *β* coefficient, summing these products, dividing the sum by the aggregate of all *β* coefficients, and multiplying by 10 to rescale the score to a 0–10 metric. For domain‐specific SDoH scores, the process was similar. Each binary SDoH within a particular domain (for instance, the financial circumstances domain, which encompasses three indicators: household income, employment status, and food security) was multiplied by its respective *β* coefficient, summed, divided by the total of all *β* coefficients, and then multiplied by 10. The overall SDoH score was derived from the cumulative sum of the five domain‐specific SDoH scores. The resulting weighted composite SDoH score ranged from 0 to 10, with higher scores indicating a greater burden of adverse SDoH [20]. Therefore, a one‐unit increase in the SDoH score represents a one‐point increase in the weighted adverse SDoH burden on the 0–10 scale.

### 2.4. Assessment of other covariates

NHANES collected a range of demographic data, which encompassed variables such as age, gender, ethnic background, marital status, educational attainment, poverty–income ratio, levels of PA, energy intake, smoking behaviors, and alcohol consumption, through household interviews. Additionally, measurements of body weight and height were acquired during physical assessments at the mobile examination center. The average energy intake was determined by calculating the mean of the two reported values from the 24‐hour dietary recall interviews. Smoking and alcohol consumption were operationalized as binary variables (indicating presence or absence). Ethnicity was classified into five distinct categories: Mexican American, Other Hispanic, Non‐Hispanic White, Non‐Hispanic Black, and Other (which includes individuals identifying as multiracial). For the purposes of subgroup analysis, race was further condensed into four categories: Mexican American, Non‐Hispanic White, Non‐Hispanic Black, and Other. Marital status was divided into three classifications: married or cohabitating, single (inclusive of widowed, divorced, or separated), and never married. Educational attainment was organized into five categories: less than 9th grade, 9–11th grade (including 12th grade without a diploma), high school graduate or equivalent (such as GED), some college or associate degree, and college graduate or higher. Self‐reported PA was converted into metabolic equivalent (MET) minutes of moderate to vigorous PA per week, following the guidelines established by the World Health Organization (WHO) [21]. PA in MET‐min/wk is determined by the equation: PA (MET − min/wk) = MET × weekly frequency × duration of each PA session. Participants were classified according to their adherence to the American PA guidelines, with those achieving less than 600 MET‐min/wk categorized as engaging in low PA, whereas those exceeding this threshold were considered to have high PA levels [22]. BMI was computed by dividing an individual′s weight in kilograms by the square of their height in meters (kg/m^2^) [23]. For the purpose of subgroup analysis, participants were divided into four distinct BMI categories: underweight (BMI < 18.5), normal weight (18.5 ≤ BMI < 25), overweight (25 ≤ BMI < 30), and obese (BMI ≥ 30). The poverty income ratio was classified into three categories: less than 1.3, between 1.3 and less than 3.5, and 3.5 or greater. Additionally, age was divided into two groups: those aged 60 years or younger and those older than 60 years.

### 2.5. Statistical analysis

The data were analyzed according to analytical guidelines, using the recommended survey weights for NHANES. Data processing and analysis were performed using R Version 4.4.0 (2024‐04‐24), along with Storm Statistical Platform (http://www.medsta.cn/software). Two‐sided statistical tests were conducted, with statistical significance set at *p* < 0.05.

Baseline characteristics were summarized using unweighted frequencies with weighted percentages for categorical variables, and weighted medians and interquartile ranges (IQRs) for continuous variables. Participants were categorized based on PD status (with or without PD). Continuous variables with a normal distribution were expressed as Mean (SE) and compared between groups using independent‐sample *t*‐tests. Categorical variables were presented as *n* (%) and compared using the chi‐square test or Fisher′s exact test. A two‐sided *p* value < 0.05 was considered statistically significant.

A logistic regression model was applied to examine the association between SDoH (either continuous or categorical) and PD. RCS models were used to evaluate the dose–response association between SDoH score and PD prevalence. The overall association and potential nonlinearity were assessed using *p* for overall and *p* for nonlinearity, respectively. The crude model was adjusted for none. Model 2 was adjusted for sex (female and male) and age (continuous). Model 3 was further adjusted for ethnic background, marital status, educational level, poverty–income ratio, PA, energy intake level, BMI (continuous), smoking habits (Yes, No), and alcohol consumption (Yes, No). Two sensitivity analyses were conducted by rerunning the fully adjusted model after excluding PD cases occurring within the first 2 years of follow‐up and by performing a complete‐case analysis that removed all participants with missing covariate data.

For subgroup analysis, SDoH was considered as a continuous variable and we stratified the data by sex, age (20–60, > 60), educational level, poverty–income ratio, PA, energy intake level, race, smoking status, PA, BMI, current alcohol consumption (Yes, No) and PD. Interaction analysis was performed by the likelihood ratio test.

## 3. Results

### 3.1. Baseline characteristics

A total of 48,637 participants were included in this study, of whom 48,311 (99.33%) were classified as non‐PD and 326 (0.67%) were classified as PD. The mean (SE) age of the participants was 46.88 (0.19) years. Significant between‐group differences (*p* < 0.05) were observed for age, PIR, PA, BMI, overall SDoH score, race, education level, SDoH–work, SDoH–ratio, SDoH–food, SDoH–education, SDoH–healthcare, SDoH–insurance, and SDoH–marital. In contrast, no statistically significant differences (*p* > 0.05) were observed for marital status, mean energy intake (two‐day average), sex, smoking history, alcohol consumption, or SDoH–instability (Table 1).

**Table 1:** Baseline characteristics of participants according to Parkinson′s disease status.

Variable	Total (*n* = 48637)	Non‐PD (*n* = 48311)	PD (*n* = 326)	Statistic	*p*
Age, mean (SE)	46.88 (0.19)	46.81 (0.19)	59.61 (1.08)	*t* = 12.14	< 0.001
Marital, mean (SE)	2.42 (0.02)	2.42 (0.02)	2.32 (0.14)	*t* = −0.78	0.434
PIR, mean (SE)	2.99 (0.03)	2.99 (0.03)	2.47 (0.12)	*t* = −4.31	< 0.001
Physical activity, mean (SE)	3411.81 (45.84)	3419.67 (46.17)	2030.26 (268.26)	*t* = −5.07	< 0.001
Mean energy intake(two‐day average), mean (SE)	2132.02 (5.94)	2132.74 (5.96)	2005.74 (66.60)	*t* = −1.90	0.060
BMI, mean (SE)	29.19 (0.06)	29.18 (0.06)	30.96 (0.45)	*t* = 3.87	< 0.001
SDoH, mean (SE)	2.30 (0.03)	2.30 (0.03)	2.90 (0.17)	*t* = 3.63	< 0.001
Sex, *n* (%)				*χ*2 = 1.63	0.255
male	23490 (48.08)	23322 (48.10)	168 (44.25)		
female	25147 (51.92)	24989 (51.90)	158 (55.75)		
Race, *n* (%)				*χ*2 = 20.42	< 0.001
Mexican American	8153 (7.86)	8119 (7.88)	34 (4.32)		
Other Hispanic	3835 (5.35)	3814 (5.36)	21 (3.72)		
Non‐Hispanic White	22074 (68.93)	21864 (68.87)	210 (80.37)		
Non‐Hispanic Black	10086 (11.02)	10036 (11.03)	50 (9.40)		
Other race (including multiracial)	4489 (6.84)	4478 (6.87)	11 (2.20)		
Education, *n* (%)				*χ*2 = 11.99	0.041
Less than 9th grade	5706 (5.71)	5664 (5.70)	42 (7.51)		
9–11th grade (including 12th grade with no diploma)	7220 (11.21)	7160 (11.20)	60 (13.82)		
High school grad/GED or equivalent	11253 (23.94)	11172 (23.92)	81 (27.05)		
Some college or AA degree	13833 (30.94)	13744 (30.93)	89 (32.16)		
College graduate or above	10625 (28.20)	10571 (28.25)	54 (19.46)		
Smoking history, *n* (%)				*χ*2 = 1.33	0.275
No	26366 (53.77)	26209 (53.79)	157 (50.32)		
Yes	22271 (46.23)	22102 (46.21)	169 (49.68)		
Alcohol consumption, *n* (%)				*χ*2 = 4.00	0.119
No	14209 (24.93)	14097 (24.90)	112 (30.13)		
Yes	34428 (75.07)	34214 (75.10)	214 (69.87)		
SDoH–work, *n* (%)				*χ*2 = 102.42	< 0.001
0	37546 (80.94)	37359 (81.07)	187 (57.04)		
1	11091 (19.06)	10952 (18.93)	139 (42.96)		
SDoH–ratio, *n* (%)				*χ*2 = 22.09	< 0.001
0	17871 (49.40)	17787 (49.48)	84 (35.27)		
1	30766 (50.60)	30524 (50.52)	242 (64.73)		
SDoH–food, *n* (%)				*χ*2 = 15.67	< 0.001
0	34881 (78.53)	34667 (78.58)	214 (68.75)		
1	13756 (21.47)	13644 (21.42)	112 (31.25)		
SDoH–education, *n* (%)				*χ*2 = 3.82	0.049
0	35711 (83.08)	35487 (83.11)	224 (78.67)		
1	12926 (16.92)	12824 (16.89)	102 (21.33)		
SDoH–healthcare, *n* (%)				*χ*2 = 35.75	< 0.001
0	39671 (82.59)	39362 (82.51)	309 (96.22)		
1	8966 (17.41)	8949 (17.49)	17 (3.78)		
SDoH–insurance, *n* (%)				*χ*2 = 21.41	< 0.001
0	26063 (63.69)	25922 (63.77)	141 (50.31)		
1	22574 (36.31)	22389 (36.23)	185 (49.69)		
SDoH–instability, *n* (%)				*χ*2 = 0.05	0.866
0	30284 (67.85)	30076 (67.85)	208 (67.21)		
1	18353 (32.15)	18235 (32.15)	118 (32.79)		
SDoH–marital, *n* (%)				*χ*2 = 6.48	0.045
0	29122 (63.51)	28954 (63.56)	168 (56.14)		
1	19515 (36.49)	19357 (36.44)	158 (43.86)		

Abbreviations: *χ* ^2^, chi‐square test; BMI, body mass index; PIR, poverty income ratio; SDoH, social determinants of health; SE, standard error; *t*, *t*‐test.

### 3.2. Associations between SDoH and PD

**Figure 2:**
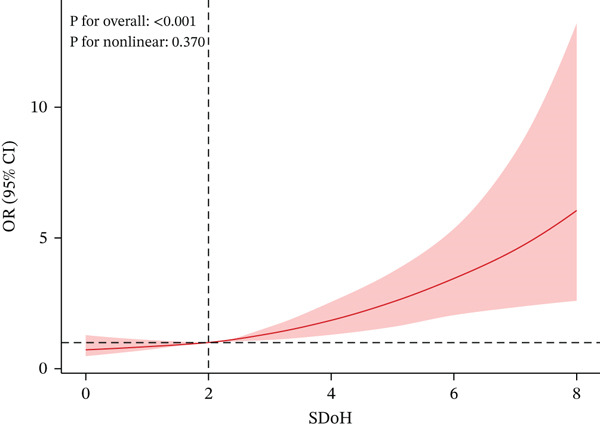
Restricted cubic spline (RCS) of the association between SDoH and the prevalence of Parkinson′s disease. RCS analysis showed no evidence of nonlinearity in the association between SDoH score and PD prevalence (*p* for nonlinearity = 0.370). Abbreviation: SDoH, social determinants of health.

In this study, logistic regression models were employed to examine the association between SDoH and the prevalence of PD. Three hierarchical models were constructed to evaluate the robustness of the association. As shown in Table 2, the crude logistic model demonstrated a statistically significant positive association between SDoH and PD (OR 1.16, 95% CI 1.08–1.26; *p* < 0.001). After adjusting for sex and age (Model 2), the association remained significant (OR 1.24, 95% CI 1.14–1.34; *p* < 0.001). In the fully adjusted model (Model 3), controlling for all listed covariates, the association was further strengthened (OR 1.31, 95% CI 1.16–1.47; *p* < 0.001; Table 2). RCS analysis showed a positive association between SDoH score and PD prevalence, with no evidence of nonlinearity (*p* for nonlinearity = 0.370; Figure 2).

**Table 2:** Association between combined and individual SDoH domains and Parkinson′s disease.

Variables	Model 1		Model 2		Model 3	
	OR (95% CI)	*p*	OR (95% CI)	*p*	OR (95% CI)	*p*
SDoH	1.16 (1.08~1.26)	< 0.001	1.24 (1.14~1.34)	< 0.001	1.31 (1.16~1.47)	< 0.001
SDoH–work	3.23 (2.41~4.32)	< 0.001	3.95 (2.94~5.30)	< 0.001	4.15 (2.70~6.40)	< 0.001
SDoH–ratio	1.80 (1.30~2.48)	< 0.001	1.75 (1.26~2.42)	0.001	0.76 (0.39~1.49)	0.419
SDoH–food	1.67 (1.23~2.27)	0.001	2.31 (1.70~3.13)	< 0.001	1.96 (1.39~2.76)	< 0.001
SDoH–education	1.33 (1.00~1.78)	0.051	1.11 (0.83~1.50)	0.480	0.93 (0.68~1.27)	0.651
SDoH–healthcare	0.19 (0.11~0.32)	< 0.001	0.31 (0.18~0.55)	< 0.001	0.27 (0.15~0.49)	< 0.001
SDoH–insurance	1.74 (1.26~2.40)	< 0.001	1.68 (1.21~2.32)	0.002	1.39 (1.00~1.94)	0.052
SDoH–instability	1.03 (0.74~1.44)	0.866	1.60 (1.15~2.24)	0.007	1.28 (0.92~1.79)	0.145
SDoH–marital	1.36 (1.01~1.85)	0.048	1.33 (0.97~1.82)	0.074	0.93 (0.62~1.39)	0.712

*Note:* Each model included both the unadjusted (Model 1) and the sex–age adjusted (Model 2) analyses. For SDoH–education, covariates included sex, race, smoking history, alcohol use, age, marital status, PIR, physical activity, mean energy intake (two‐day average), and BMI. For all other domains and combined SDoH, the model additionally incorporated the variable education level (cultural attainment). For the continuous SDoH score, ORs represent the odds of PD prevalence per one‐point increase in the weighted adverse SDoH burden score on the 0–10 scale.

Abbreviations: BMI, body mass index; CI, confidence interval; OR, odds ratio; PIR, poverty income ratio; SDoH, social determinants of health.

Strong positive associations were found for the work, food, ratio, and insurance domains, which remained significant or increased in magnitude after adjustment. Education and marital status showed weaker or borderline associations that lost significance after adjustment. The healthcare domain demonstrated a clear protective effect, while the instability domain became significantly associated with PD after adjustment. For the continuous SDoH score, ORs were interpreted per one‐point increase in the weighted adverse SDoH burden score on the 0–10 scale (Table 2). The findings from both sensitivity analyses were consistent with the primary results (Tables S1 and S2).

### 3.3. Subgroup analysis and sensitivity analysis

The association between SDoH and the prevalence of PD remained stable across multiple subgroup analyses. Specifically, in stratifications by sex, smoking history, alcohol consumption, race, marital status, and poverty income ratio, the SDoH was consistently and significantly associated with increased PD prevalence. Furthermore, there were no statistically significant differences between subgroups (*p* for interaction > 0.05). Significant heterogeneity in associations was observed across stratifications by BMI (*p* = 0.033) and customized age grouping (*p* < 0.001). The age‐related interaction was particularly pronounced: the association between SDoH and PD prevalence was strongest among middle‐aged participants, whereas no significant association was detected in the older age group. No significant associations between SDoH and PD prevalence were observed in several subgroups, including individuals with low PA, extremely low BMI, certain racial subgroups, unmarried individuals, and the older population (Table 3).

**Table 3:** Subgroup analysis of the association between SDOH and the prevalence of Parkinson′s disease.

Subgroup	*n* (%)	OR (95% CI)	*p*	*p* for interaction
All patients	48637 (100.00)	1.31 (1.16~1.47)	< 0.001	
Sex				0.442
male	23490 (48.30)	1.39 (1.18~1.65)	< 0.001	
female	25147 (51.70)	1.26 (1.10~1.44)	0.001	
Education				0.161
Less than 9th grade	5706 (11.73)	1.08 (0.79~1.48)	0.624	
9–11th grade (including 12th grade with no diploma)	7220 (14.84)	1.35 (1.07~1.71)	0.013	
High school grad/GED or equivalent	11253 (23.14)	1.54 (1.28~1.84)	< 0.001	
Some college or AA degree	13833 (28.44)	1.18 (0.96~1.44)	0.121	
College graduate or above	10625 (21.85)	1.31 (1.01~1.70)	0.041	
Smoking history, *n* (%)				0.106
No	26366 (54.21)	1.30 (1.09~1.54)	0.004	
Yes	22271 (45.79)	1.31 (1.13~1.52)	< 0.001	
Alcohol consumption, *n* (%)				0.940
No	14209 (29.21)	1.30 (1.08~1.56)	0.006	
Yes	34428 (70.79)	1.32 (1.16~1.50)	< 0.001	
Physical activity				0.050
Low physical activity	15865 (32.62)	1.14 (0.96~1.35)	0.143	
High physical activity	32772 (67.38)	1.46 (1.25~1.70)	< 0.001	
BMI				0.033
< 18.5	726 (1.49)	7.74 (0.27~222.38)	0.235	
18.5–25	12882 (26.49)	1.31 (1.00~1.70)	0.048	
25–30	15361 (31.58)	1.36 (1.14~1.64)	0.001	
≥ 30	19668 (40.44)	1.31 (1.12~1.53)	< 0.001	
Race				0.466
Mexican American	8153 (16.76)	1.37 (1.01~1.86)	0.044	
Non‐Hispanic White	22074 (45.39)	1.28 (1.12~1.46)	< 0.001	
Non‐Hispanic Black	10086 (20.74)	1.52 (1.21~1.90)	< 0.001	
Other	8324 (17.11)	1.36 (0.90~2.06)	0.147	
Marital				0.095
Married or living with a partner	29122 (59.88)	1.49 (1.26~1.76)	< 0.001	
Single (widowed/divorced/separated)	11068 (22.76)	1.15 (0.94~1.40)	0.168	
Never married	8447 (17.37)	1.67 (1.29~2.16)	< 0.001	
PIR				0.063
< 1.3	15178 (31.21)	1.42 (1.19~1.68)	< 0.001	
1.3 to < 3.5	18472 (37.98)	1.25 (1.06~1.48)	0.011	
≥ 3.5	14987 (30.81)	1.37 (1.04~1.81)	0.027	
Age				< 0.001
≤60	32107 (66.01)	1.35 (1.13~1.61)	< 0.001	
>60	16530 (33.99)	1.14 (0.95~1.37)	0.162	

Abbreviations: BMI, body mass index; CI, confidence interval; OR, odds ratio; PIR, poverty income ratio.

## 4. Discussion

Our study, utilizing nationally representative data from NHANES 2001–2020, provides a comprehensive evaluation of the association between SDoH and the prevalence of PD among US adults. Three major findings emerged. First, the burden of cumulative adverse SDoH was significantly associated with higher PD prevalence, and RCS analysis showed no evidence of nonlinearity in this association. Second, the specific domains accounted differentially for this prevalence; unemployment and food insecurity emerged as strong prevalence factors, whereas access to health care exhibited a complex pattern of association. Third, this relationship was significantly effect‐modified by age, with strong SDoH–PD relationship observed among middle‐aged adults (60 years), whereas weakening in the elderly. These results highlight the importance of taking into account social factors in the epidemiologic understanding and prevention of PD. It is also reassuring that the consistency of the results in the different sensitivity analyses lends validity to our main results.

These hypothesized pathways whereby SDoH influence PD prevalence may be intricate and operate “through the social‐biological‐psychological pathway.” Our results for socioeconomic status (poverty–income ratio) and food insecurity do align with the hypothesis of “resource deprivation” because food insecurity and deprivation exacerbate poverty conditions that may restrict access to high‐value foods with brain protective content (e.g., antioxidants, Omega‐3 fatty acids). These have all the potential to increase the oxidative stress and damage the mitochondrial functioning, which are primary drivers of the apoptotic process of dopaminergic neurons [24, 25]. Recently published a comprehensive review confirmed that dietary consumption patterns poor in antioxidant content could increase the prevalence of PD by substantial levels of statistical significance. Social and economic conditions of food access can be implicated directly to an increase in the biological vulnerability of susceptibility [26]. Low diet quality due to poverty means not just low intake of antioxidants, but could also mean gut dysbiosis. Recent breakthrough studies imply that pro‐inflammatory diets can break the intestinal barrier, activate abnormal aggregation of *α*‐synuclein in the enteric nervous system which may spread to the substantia nigra via vagus nerve (Braak hypothesis) [27, 28]. Dietarily, individuals who experience food insecurity commonly eat less expensive pro‐inflammatory foods that may promote increased pathological activities within this prodromal phase of PD. Financial stress, however, is also a long term psychosocial prevalence factor that can cause the hypothalamic‐pituitary‐adrenal axis to be dysregulated. Sustained glucocorticoid release and secondary systemic inflammation could damage the blood‐brain barrier and lead to neuroinflammation and facilitate neurodegeneration [29, 30]. A recent review points to the fact that the ongoing activation of the peripheral immune system is an important mediator of dopaminergic neuronal death [31]. Hence, adverse SDoH may plausibly contribute to biological vulnerability, although causal inference cannot be established in the present cross‐sectional study.

By contrast, the surprising relationship to healthcare access, in which the reported absence of healthcare access seems to be correlated with lesser prevalence of PD, likely represents the mediating effect of ascertainment bias far more so than a true protective effect [32]. Those individuals with persistent access to healthcare have access to appropriate management of PD comorbidities (i.e., diabetes, hypertension) and systematic screening for prodromal symptomology (e.g., psychiatric or gait symptomology). This class of susceptible populations is experiencing tremor, bradykinesia, and thus, experiences early detection and treatment [33]. In other words, without resources like this, disease diagnosis is masked. These susceptible populations may never even be diagnosed due to competing prevalences, cardiovascular disease, which creates an artificial “low prevalence” in the data. Our results thus reflect structural inequalities in the healthcare system: those most socially pushed prevalence would end up least likely being picked up in clinical screening. Noteworthily, stratified analyses showed that this SDoH–PD association was subject to substantial effect modification by age. The strong correlation among the middle‐aged (≤ 60 years), together, indicates that the middle aged‐range “might be a critical window for exposure” where the social exposure critically interacts with the genetic prevalence (i.e., biological weakness [e.g., *α*synuclein accumulation]) to trigger the disease progression. On the contrary, the weaker association for advanced age could be due to “survivor bias,” that is, less socially privileged people die of other competing deaths before developing PD, or because aging related biological mechanisms rather than social ones gain the upper hand at very old age [6, 34].

This finding comports well with, and our study expands, the prior literature linking low SES with heightened PD prevalence [12, 35]. Our findings replicate and extend prior literature in this respect and are particularly innovative as we identify eight distinct SDoH domains from which to target intervention, rather than broad SES categories or at single‐attribute categories such as insurance coverage, distinguishing this study from those that use single SES constructs. Additionally, our results of healthcare resource protection agrees with the result of Gross et al. [33], who showed medical care‐use strongly affected PD case ascertainment and outcome. This is due to the use of a national sample, and a weighted SDoH score, this study′s findings are more specific, yet generalizable than previous local studies and brings a new point of reference for SD based prevalence stratification.

There are several strengths in our study that increases the validity and applicability of our findings. Using ten consecutive cycles of NHANES (2001–2020) with a robust multistage probability sampling design, we acquired a nationally representative sample of US adults. Our PD ascertainment was based on a reproducible medication‐based approach that has been used in previous NHANES‐based PD studies. This approach enabled PD status to be consistently defined across multiple NHANES cycles in the absence of a clinician‐adjudicated PD diagnosis variable [36, 37]. SDoH domains were delineated based upon the standardized Healthy People 2030 taxonomy18 and was therefore possible to define all domains including economic, education, healthcare, and neighborhood in an integrated manner. The present study also transcends typical socioeconomic measurements by defining more specific actionable domains that could be considered tangible targets of a prevention and/or policy interventions, for example in terms of food security or healthcare access. However, several limitations should be considered. As NHANES is cross‐sectional, a causal analysis cannot be conducted and residual confounding by unmeasured genetic or environmental factors remain possible. The observed associations should not be interpreted as evidence of causality or incident PD risk, and reverse causation is possible. Therefore, our findings should be interpreted as prevalence associations, and longitudinal studies are needed to evaluate temporality and incident PD. The measures of SDoH were only obtained at baseline and may not reflect cumulative or time‐varying exposures. PD ascertainment based on self‐reported antiparkinsonian medication use is a key limitation. Although this approach has been used in prior NHANES‐based PD studies, it is not equivalent to clinician‐adjudicated diagnosis and may lead to misclassification, including missed untreated or early‐stage cases and inclusion of participants using related medications for other indications. Therefore, our findings should be interpreted as associations with NHANES‐ascertained PD prevalence rather than clinically confirmed incident PD. The variables on SDoH were obtained via self‐reports and, therefore, they were susceptible to recall bias. Second, we did not consider the interactions between SDoH domains, potentially overstating simplicity of social disadvantage. Third, generalizability to the United States may be limited by the differences across countries in terms of social structure and health care.

## 5. Conclusion

Poor SDoH are independently associated with PD prevalence in middle‐aged adults. Adverse social determinants were significantly associated with higher prevalence of PD, particularly among individuals aged 60 years and younger, and RCS analysis showed no evidence of nonlinearity. Unemployment and food insecurity showed strong positive associations with PD prevalence, whereas the inverse association with healthcare access likely reflects ascertainment bias rather than a true protective effect. These findings highlight the need to incorporate social determinant assessments into PD screening and epidemiologic research. Future longitudinal studies are needed to clarify temporality and establish causal inferences regarding social determinants and incident PD.

## Funding

This study was supported by Boyan Fang, No. 2022YFC3602603, 2020‐069; and Detao Meng, 2023R‐03.

## Ethics Statement

Ethics approval is unnecessary as NHANES is a public database with prior ethical approval for participants, allowing researchers to access data for scholarly use.

## Conflicts of Interest

The authors declare no conflicts of interest.

## Supporting information


**Supporting Information** Additional supporting information can be found online in the Supporting Information section. Table S1: Association between combined and individual SDoH domains and PD using complete‐case analysis by excluding all participants with missing covariate values. Table S2: Association between combined and individual SDoH domains and PD excluding all PD cases that occurred within the first 2 years of follow‐up.

## Data Availability

TThe data that support the findings of this study are available in National Health and Nutrition Examination Survey (HANES) at https://wwwn.cdc.gov/nchs/nhanes/, reference number NHANES 2001–2020. These data were derived from the following resources available in the public domain: ‐ U.S. National Center for Health Statistics, https://wwwn.cdc.gov/nchs/nhanes/.

## References

[1] Dorsey E. R. , Elbaz A. , Nichols E. , Abbasi N. , Abd-Allah F. , Abdelalim A. , Adsuar J. C. , Ansha M. G. , Brayne C. , Choi J. Y. J. , Collado-Mateo D. , Dahodwala N. , do H. P. , Edessa D. , Endres M. , Fereshtehnejad S. M. , Foreman K. J. , Gankpe F. G. , Gupta R. , Hamidi S. , Hankey G. J. , Hay S. I. , Hegazy M. I. , Hibstu D. T. , Kasaeian A. , Khader Y. , Khalil I. , Khang Y. H. , Kim Y. J. , Kokubo Y. , Logroscino G. , Massano J. , Mohamed Ibrahim N. , Mohammed M. A. , Mohammadi A. , Moradi-Lakeh M. , Naghavi M. , Nguyen B. T. , Nirayo Y. L. , Ogbo F. A. , Owolabi M. O. , Pereira D. M. , Postma M. J. , Qorbani M. , Rahman M. A. , Roba K. T. , Safari H. , Safiri S. , Satpathy M. , Sawhney M. , Shafieesabet A. , Shiferaw M. S. , Smith M. , Szoeke C. E. I. , Tabarés-Seisdedos R. , Truong N. T. , Ukwaja K. N. , Venketasubramanian N. , Villafaina S. , weldegwergs K. , Westerman R. , Wijeratne T. , Winkler A. S. , Xuan B. T. , Yonemoto N. , Feigin V. L. , Vos T. , and Murray C. J. L. , Global, Regional, and National Burden of Parkinson′s Disease, 1990–2016: A Systematic Analysis for the Global Burden of Disease Study 2016, Lancet Neurology. (2018) 17, no. 11, 939–953, 10.1016/S1474-4422(18)30295-3, 30287051. 30287051 PMC6191528

[2] Su D. , Cui Y. , He C. , Yin P. , Bai R. , Zhu J. , Lam J. S. T. , Zhang J. , Yan R. , Zheng X. , Wu J. , Zhao D. , Wang A. , Zhou M. , and Feng T. , Projections for Prevalence of Parkinson′s Disease and Its Driving Factors in 195 Countries and Territories to 2050: Modelling Study of Global Burden of Disease Study 2021, BMJ. (2025) 388, e080952, 10.1136/bmj-2024-080952, 40044233. 40044233 PMC11881235

[3] Atterling Brolin K. , Schaeffer E. , Kuri A. , Rumrich I. K. , Schumacher Schuh A. F. , Darweesh S. K. L. , Kaasinen V. , Tolppanen A. M. , Chahine L. M. , and Noyce A. J. , Environmental Risk Factors for Parkinson′s Disease: A Critical Review and Policy Implications, Movement Disorders. (2025) 40, no. 2, 204–221, 10.1002/mds.30067, 39601461. 39601461 PMC11832802

[4] Lüth T. , Caliebe A. , Gabbert C. , Sendel S. , Laabs B. H. , König I. R. , Klein C. , and Trinh J. , Longitudinal Assessment of the Association Between Pesticide Exposure and Lifestyle With Parkinson′s Disease Motor Severity, npj Parkinson′s Disease. (2025) 11, no. 1, 10.1038/s41531-025-01010-2, 40506444. PMC12162858 40506444

[5] Gómez C. A. , Kleinman D. V. , Pronk N. , Wrenn Gordon G. L. , Ochiai E. , Blakey C. , Johnson A. , and Brewer K. H. , Addressing Health Equity and Social Determinants of Health Through Healthy People 2030, Journal of Public Health Management and Practice. (2021) 27, no. 6 Supplement, S249–S257, 10.1097/PHH.0000000000001297, 33729197. 33729197 PMC8478299

[6] Bundy J. D. , Mills K. T. , He H. , LaVeist T. A. , Ferdinand K. C. , Chen J. , and He J. , Social Determinants of Health and Premature Death Among Adults in the USA From 1999 to 2018: A National Cohort Study, Lancet Public Health. (2023) 8, no. 6, e422–e431, 10.1016/S2468-2667(23)00081-6, 37244672. 37244672 PMC10349537

[7] Braveman P. and Gottlieb L. , The Social Determinants of Health: It′s Time to Consider the Causes of the Causes, Public Health Reports. (2014) 129, no. 2 Supplement, 19–31, 10.1177/00333549141291S206, 24385661. PMC3863696 24385661

[8] Zhong J. , Zhang Y. , Zhu K. , Li R. , Zhou X. , Yao P. , Franco O. H. , Manson J. A. E. , Pan A. , and Liu G. , Associations of Social Determinants of Health With Life Expectancy and Future Health Risks Among Individuals With Type 2 Diabetes: Two Nationwide Cohort Studies in the UK and USA, Lancet Healthy Longevity. (2024) 5, no. 8, e542–e551, 10.1016/S2666-7568(24)00116-8, 39106873. 39106873

[9] Zhu R. , Wang R. , He J. , Wang L. , Chen H. , Niu X. , Sun Y. , Guan Y. , Gong Y. , Zhang L. , An P. , Li K. , Ren F. , Xu W. , and Guo J. , Prevalence of Cardiovascular-Kidney-Metabolic Syndrome Stages by Social Determinants of Health, JAMA Network Open. (2024) 7, no. 11, e2445309, 10.1001/jamanetworkopen.2024.45309, 39556396. 39556396 PMC11574692

[10] Zhang L. , Reshetnyak E. , Ringel J. B. , Pinheiro L. C. , Carson A. , Cummings D. M. , Durant R. W. , Malla G. , and Safford M. M. , Social Determinants of Health and Cardiovascular Prevalence Among Adults With Diabetes: The Reasons for Geographic and Racial Differences in Stroke (REGARDS) Study, Diabetes & Metabolism Journal. (2024) 48, 1073–1083. 39034653 10.4093/dmj.2023.0380 PMC11621655

[11] Yoon S. Y. , Shin J. , Chang J. S. , Lee S. C. , and Kim Y. W. , Effects of Socioeconomic Status on Mortality After Parkinson′s Disease: A Nationwide Population-Based Matched Cohort Study in Korean Populations, Parkinsonism & Related Disorders. (2020) 80, 206–211. 33129703 10.1016/j.parkreldis.2020.10.017

[12] Najafi F. , Mansournia M. A. , Abdollahpour I. , Rohani M. , Vahid F. , and Nedjat S. , Association Between Socioeconomic Status and Parkinson’s Disease: Findings From a Large Incident Case–Control Study, BMJ Neurology Open. (2023) 5, e000386, 10.1136/bmjno-2022-000386. PMC9933671 36817512

[13] Towfighi A. , Berger R. P. , Corley A. M. , Glymour M. M. , Manly J. J. , and Skolarus L. E. , Recommendations on Social Determinants of Health in Neurologic Disease, Neurology. (2023) 101, S26–S17, 10.1212/WNL.000000000020756. PMC12345359 37580147

[14] Ke L. , Zhao L. , Xing W. , and Tang Q. , Association Between Parkinson′s Disease and Cardiovascular Disease Mortality: A Prospective Population-Based Study From NHANES, Lipids in Health and Disease. (2024) 23, no. 1, 10.1186/s12944-024-02200-2, 38965560. PMC11223358 38965560

[15] Fox S. H. , Katzenschlager R. , Lim S. Y. , Ravina B. , Seppi K. , Coelho M. , Poewe W. , Rascol O. , Goetz C. G. , and Sampaio C. , The Movement Disorder Society Evidence-Based Medicine Review Update: Treatments for the Motor Symptoms of Parkinson′s Disease, Movement Disorders. (2011) 26, no. S3, S2–S41, 10.1002/mds.23829, 22021173. 22021173

[16] Botelho J. , Lyra P. , Proença L. , Godinho C. , Mendes J. J. , and Machado V. , Relationship Between Blood and Standard Biochemistry Levels With Periodontitis in Parkinson′s Disease Patients: Data From the NHANES 2011–2012, Journal of Personalized Medicine. (2020) 10, no. 3, 10.3390/jpm10030069, 32722393. PMC7565163 32722393

[17] Li J. , Lei L. , Wang W. , Ding W. , Yu Y. , Pu B. , Peng Y. , Li Y. , Zhang L. , and Guo Y. , Social Risk Profile and Cardiovascular-Kidney-Metabolic Syndrome in US Adults, Journal of the American Heart Association. (2024) 13, no. 16, e034996, 10.1161/JAHA.124.034996, 39136302. 39136302 PMC11963957

[18] US-Department-of-Health-and-Human-Services , Healthy People 2030, 2023, Social Determinants of Health, https://health.gov/healthypeople/priority-areas/social-determinants-health.

[19] Huang H. , Wei T. , Huang Y. , Zhang A. , Zhang H. , Zhang Z. , Xu Y. , Pan H. , Kong L. , Li Y. , and Li F. , Association Between Social Determinants of Health and Survival Among the US Cancer Survivors Population, BMC Medicine. (2024) 22, no. 1, 10.1186/s12916-024-03563-0, 39183305. PMC11346002 39183305

[20] Yang Q. , Shi P. , Pan L. , and Huang Z. , Social Determzinants of Health (SDOH) Associated With the Risk of All-Cause Mortality and Life Expectancy in US Adults With Cardiovascular-Kidney-Metabolic Syndrome: a NHANES 2001–2018 Cohort Study, BMC Cardiovascular Disorders. (2025) 25, no. 1, 10.1186/s12872-025-04812-7, 40375145. PMC12079812 40375145

[21] World-Health-Organization , Global Physical Activity Questionnaire (GPAQ) Analysis Guide, https://www.who.int/docs/default-source/ncds/ncd-surveillance/gpaq-analysis-guide.pdf.

[22] Piercy K. L. , Troiano R. P. , Ballard R. M. , Carlson S. A. , Fulton J. E. , Galuska D. A. , George S. M. , and Olson R. D. , The Physical Activity Guidelines for Americans, JAMA. (2018) 320, no. 19, 2020–2028, 10.1001/jama.2018.14854, 30418471. 30418471 PMC9582631

[23] Aggarwal R. , Ostrominski J. W. , and Vaduganathan M. , Prevalence of Cardiovascular-Kidney-Metabolic Syndrome Stages in US Adults, 2011-2020, JAMA. (2024) 331, no. 21, 1858–1860, 10.1001/jama.2024.6892, 38717747. 38717747 PMC11079779

[24] Seidl S. E. , Santiago J. A. , Bilyk H. , and Potashkin J. A. , The Emerging Role of Nutrition in Parkinson′s Disease, Frontiers in Aging Neuroscience. (2014) 6, 74578, 10.3389/fnagi.2014.00036, 24639650. PMC3945400 24639650

[25] Cannas C. , Lostia G. , Serra P. A. , Peana A. T. , and Migheli R. , Food and Food Waste Antioxidants: Could They Be a Potent Defence Against Parkinson′s Disease?, Antioxidants. (2024) 13, no. 6, 10.3390/antiox13060645, 38929084. PMC11200518 38929084

[26] Akhlaghi M. , Foshati S. , Hashemi Moghaddam F. , Sasani M. R. , and Kazemi A. , Foods and Dietary Intakes and the Risk of Parkinson’s Disease: A Systematic Review and Dose–Response Meta-analysis of Prospective Cohort Studies, Nutrition Reviews. (2025) nuaf224, 10.1093/nutrit/nuaf224. 41252180

[27] Scheperjans F. , Aho V. , Pereira P. A. B. , Koskinen K. , Paulin L. , Pekkonen E. , Haapaniemi E. , Kaakkola S. , Eerola-Rautio J. , Pohja M. , Kinnunen E. , Murros K. , and Auvinen P. , Gut Microbiota Are Related to Parkinson′s Disease and Clinical Phenotype, Movement Disorders. (2015) 30, no. 3, 350–358, 10.1002/mds.26069, 25476529. 25476529

[28] Sampson T. R. , Debelius J. W. , Thron T. , Janssen S. , Shastri G. G. , Ilhan Z. E. , Challis C. , Schretter C. E. , Rocha S. , Gradinaru V. , Chesselet M. F. , Keshavarzian A. , Shannon K. M. , Krajmalnik-Brown R. , Wittung-Stafshede P. , Knight R. , and Mazmanian S. K. , Gut Microbiota Regulate Motor Deficits and Neuroinflammation in a Model of Parkinson′s Disease, Cell. (2016) 167, no. 6, 1469–1480, 10.1016/j.cell.2016.11.018, 27912057. 27912057 PMC5718049

[29] Hemmerle A. M. , Herman J. P. , and Seroogy K. B. , Stress, Depression and Parkinson′s Disease, Experimental Neurology. (2012) 233, no. 1, 79–86, 10.1016/j.expneurol.2011.09.035, 22001159. 22001159 PMC3268878

[30] Pajares M. , Rojo I. A. , Manda G. , Boscá L. , and Cuadrado A. , Inflammation in Parkinson′s Disease: Mechanisms and Therapeutic Implications, Cells. (2020) 9, no. 7, 10.3390/cells9071687, 32674367. PMC7408280 32674367

[31] Tansey M. G. , Wallings R. L. , Houser M. C. , Herrick M. K. , Keating C. E. , and Joers V. , Inflammation and Immune Dysfunction in Parkinson Disease, Nature Reviews Immunology. (2022) 22, no. 11, 657–673, 10.1038/s41577-022-00684-6, 35246670. PMC8895080 35246670

[32] Aamodt W. W. , Willis A. W. , and Dahodwala N. , Racial and Ethnic Disparities in Parkinson Disease: A Call to Action, Neurology: Clinical Practice. (2023) 13, no. 2, e200138, 10.1212/CPJ.0000000000200138, 37064587. 37064587 PMC10101714

[33] Gross A. , Racette B. A. , Camacho-Soto A. , Dube U. , and Searles Nielsen S. , Use of Medical Care Biases Associations Between Parkinson Disease and Other Medical Conditions, Neurology. (2018) 90, no. 24, e2155–e2165, 10.1212/WNL.0000000000005678, 29743207. 29743207 PMC5996836

[34] Collier T. J. , Kanaan N. M. , and Kordower J. H. , Aging and Parkinson′s Disease: Different Sides of the Same Coin?, Movement Disorders. (2017) 32, 983–990, 10.1002/mds.27037. 28520211 PMC5844262

[35] Yang F. , Johansson A. L. V. , Pedersen N. L. , Fang F. , Gatz M. , and Wirdefeldt K. , Socioeconomic Status in Relation to Parkinson′s Disease risk and mortality, Medicine. (2016) 95, no. 30, e4337, 10.1097/MD.0000000000004337, 27472716. 27472716 PMC5265853

[36] Jain S. , Himali J. , Beiser A. , Ton T. G. N. , Kelly-Hayes M. , Biggs M. L. , Delaney J. A. C. , Rosano C. , Seshadri S. , and Frank S. A. , Validation of Secondary Data Sources to Identify Parkinson Disease Against Clinical Diagnostic Criteria, American Journal of Epidemiology. (2015) 181, no. 3, 185–190, 10.1093/aje/kwu326, 25550359. 25550359 PMC4312428

[37] Peterson B. J. , Rocca W. A. , Bower J. H. , Savica R. , and Mielke M. M. , Identifying Incident Parkinson′s Disease Using Administrative Diagnostic Codes: A Validation Study, Clinical Parkinsonism & Related Disorders. (2020) 3, 100061, 10.1016/j.prdoa.2020.100061, 34164614. 34164614 PMC8218579

