# Supplementary material for: Social Determinants of Health and Their Association With Parkinson′s Disease Prevalence in US Adults: Insights From NHANES 2001–2020
Source: Behav Neurol. 2026 Jun 24;2026:3745561. doi: 10.1155/bn/3745561 (PMC13291721; doi:10.1155/bn/3745561)
Supplement: Supplementary file 1 — Supporting Information Additional supporting information can be found online in the Supporting Information section. Table S1: Association between combined and individual SDoH domains and PD using complete‐case analysis by excluding all participants with missing covariate values. Table S2: Association between combined and individual SDoH domains and PD excluding all PD cases that occurred within the first 2 years of follow‐up. [file BN-2026-3745561-s001.docx]

Supplementary table 1 Association between combined and individual SDoH domains and Parkinson’s Disease

| Variables | Model1 | |  | Model2 | |  | Model3 | |
| --- | --- | --- | --- | --- | --- | --- | --- | --- |
|  | OR (95%CI) | *P* |  | OR (95%CI) | *P* |  | OR (95%CI) | *P* |
| SDoH | 1.16 (1.08 ~ 1.26) | <0.001 |  | 1.24 (1.14 ~ 1.34) | <0.001 |  | 1.27 (1.09 ~ 1.49) | 0.003 |
| SDoH work | 3.23 (2.41 ~ 4.32) | <0.001 |  | 3.95 (2.94 ~ 5.30) | <0.001 |  | 2.92 (2.02 ~ 4.23) | <0.001 |
| SDoH ratio | 1.80 (1.30 ~ 2.48) | <0.001 |  | 1.75 (1.26 ~ 2.42) | 0.001 |  | 0.68 (0.25 ~ 1.84) | 0.451 |
| SDoH food | 1.67 (1.23 ~ 2.27) | 0.001 |  | 2.31 (1.70 ~ 3.13) | <0.001 |  | 1.89 (1.18 ~ 3.02) | 0.009 |
| SDoH education | 1.33 (1.00 ~ 1.78) | 0.051 |  | 1.11 (0.83 ~ 1.50) | 0.480 |  | 1.04 (0.66 ~ 1.65) | 0.871 |
| SDoH healthcare | 0.19 (0.11 ~ 0.32) | <0.001 |  | 0.31 (0.18 ~ 0.55) | <0.001 |  | 0.35 (0.17 ~ 0.74) | 0.006 |
| SDoH insurance | 1.74 (1.26 ~ 2.40) | <0.001 |  | 1.68 (1.21 ~ 2.32) | 0.002 |  | 1.72 (1.12 ~ 2.63) | 0.014 |
| SDoH instability | 1.03 (0.74 ~ 1.44) | 0.866 |  | 1.60 (1.15 ~ 2.24) | 0.007 |  | 1.37 (0.89 ~ 2.10) | 0.150 |
| SDoH Marital | 1.36 (1.01 ~ 1.85) | 0.048 |  | 1.33 (0.97 ~ 1.82) | 0.074 |  | 0.68 (0.39 ~ 1.21) | 0.195 |
| Note：We conducted a complete-case analysis by excluding all participants with missing covariate values at the inclusion stage (i.e., no imputation) and reran the fully adjusted model. Each model included both the unadjusted (Model 1) and the sex–age adjusted (Model 2) analyses. For SDOH–education, covariates included sex, race, smoking history, alcohol use, age, marital status, PIR, physical activity, mean energy intake (two-day average), and BMI.For all other domains and combined SDoH, the model additionally incorporated the variable education level (cultural attainment).  Abbreviations: BMI:body mass index, CI: Confidence Interval, OR: Odds Ratio, PIR:poverty income ratio, SDoH: social determinants of health. | | | | | | | | |

Supplementary table 2 Association between combined and individual SDoH domains and Parkinson’s Disease

| Variables | Model1 | |  | Model2 | |  | Model3 | |
| --- | --- | --- | --- | --- | --- | --- | --- | --- |
|  | OR (95%CI) | *P* |  | OR (95%CI) | *P* |  | OR (95%CI) | *P* |
| SDoH | 1.13 (1.04 ~ 1.23) | 0.005 |  | 1.21 (1.11 ~ 1.31) | <0.001 |  | 1.21 (1.03 ~ 1.41) | 0.021 |
| SDoH work | 2.93 (2.15 ~ 3.99) | <0.001 |  | 3.55 (2.59 ~ 4.85) | <0.001 |  | 2.52 (1.75 ~ 3.62) | <0.001 |
| SDoH ratio | 1.60 (1.13 ~ 2.26) | 0.009 |  | 1.57 (1.11 ~ 2.23) | 0.012 |  | 0.47 (0.19 ~ 1.20) | 0.120 |
| SDoH food | 1.57 (1.14 ~ 2.16) | 0.007 |  | 2.19 (1.59 ~ 3.00) | <0.001 |  | 1.95 (1.22 ~ 3.12) | 0.007 |
| SDoH education | 1.28 (0.95 ~ 1.71) | 0.102 |  | 1.08 (0.80 ~ 1.46) | 0.617 |  | 0.92 (0.59 ~ 1.41) | 0.691 |
| SDoH healthcare | 0.21 (0.12 ~ 0.37) | <0.001 |  | 0.36 (0.20 ~ 0.64) | <0.001 |  | 0.38 (0.18 ~ 0.80) | 0.012 |
| SDoH insurance | 1.60 (1.13 ~ 2.27) | 0.008 |  | 1.58 (1.12 ~ 2.24) | 0.011 |  | 1.50 (0.98 ~ 2.29) | 0.064 |
| SDoH instability | 1.01 (0.71 ~ 1.45) | 0.945 |  | 1.61 (1.13 ~ 2.31) | 0.010 |  | 1.48 (0.98 ~ 2.25) | 0.068 |
| SDoH Marital | 1.20 (0.86 ~ 1.66) | 0.288 |  | 1.16 (0.83 ~ 1.62) | 0.385 |  | 0.61 (0.35 ~ 1.07) | 0.090 |
| Note：We conducted a complete-case analysis by excluding all PD cases that occurred within the first two years of follow-up and reran the fully adjusted model. Each model included both the unadjusted (Model 1) and the sex–age adjusted (Model 2) analyses. For SDOH–education, covariates included sex, race, smoking history, alcohol use, age, marital status, PIR, physical activity, mean energy intake (two-day average), and BMI.For all other domains and combined SDoH, the model additionally incorporated the variable education level (cultural attainment).  Abbreviations: BMI:body mass index, CI: Confidence Interval, OR: Odds Ratio, PIR:poverty income ratio, SDoH: social determinants of health. | | | | | | | | |
